# Advantages of Fibrin Polymerization Method without the Use of Exogenous Thrombin for Vascular Tissue Engineering Applications

**DOI:** 10.3390/biomedicines10040789

**Published:** 2022-03-28

**Authors:** Vera G. Matveeva, Evgenia A. Senokosova, Viktoriia V. Sevostianova, Mariam Yu. Khanova, Tatiana V. Glushkova, Tatiana N. Akentieva, Larisa V. Antonova, Leonid S. Barbarash

**Affiliations:** Department of Experimental Medicine, Research Institute for Complex Issues of Cardiovascular Diseases, 6 Sosnovyy Boulevard, 650002 Kemerovo, Russia; matvvg@kemcardio.ru (V.G.M.); sergea@kemcardio.ru (E.A.S.); hanomu@kemcardio.ru (M.Y.K.); glushtv@kemcardio.ru (T.V.G.); akentn@kemcardio.ru (T.N.A.); antolv@kemcardio.ru (L.V.A.); reception@kemcardio.ru (L.S.B.)

**Keywords:** autologous fibrin, fibrin matrix, vascular tissue engineering, endogenous thrombin, exogenous thrombin

## Abstract

Fibrin is widely used in vascular tissue engineering. Typically, fibrin polymerization is initiated by adding exogenous thrombin. In this study, we proposed a protocol for the preparation of completely autologous fibrin without the use of endogenous thrombin and compared the properties of the prepared fibrin matrix with that obtained by the traditional method. Fibrinogen was obtained by ethanol precipitation followed by fibrin polymerization by adding either exogenous thrombin and calcium chloride (ExThr), or only calcium chloride (EnThr). We examined the structure, mechanical properties, thrombogenicity, degradation rate and cytocompatibility of fibrin matrices. Factor XIII (FXIII) quantitative assay was performed by ELISA, and FXIII activity was assessed by SDS-PAGE detection of γ-γ cross-links. The results show that network structure of EnThr fibrin was characterized by thinner fibers. The EnThr fibrin matrices had higher strength, stiffness and resistance to proteolytic degradation compared to ExThr fibrin. EnThr fibrin matrices exhibited less thrombogenicity in vitro than ExThr, and retained high cytocompatibility. Thus, the proposed approach has several advantages over the traditional method, namely the fabrication of a completely autologous coating material that has better mechanical properties, higher resistance to proteolysis and lower thrombogenicity.

## 1. Introduction

Cardiovascular diseases remain the leading cause of death worldwide [1,2]. Coronary artery atherosclerosis is the most common form of cardiovascular disease requiring an integrated approach to treatment. High grade occlusions of coronary artery are revascularized by coronary artery bypass grafting using autologous artery and vein grafts [3]. However, 30% of patients requiring vascular reconstruction do not have autologous vessels suitable for implantation [4,5]. In this case, vascular prostheses are the only alternative to autologous vessels.

Synthetic vascular prostheses based on polytetrafluoroethylene, polyethylene terephthalate and polyurethane are successfully used for replacement of large diameter vessels (>6 mm) [6]. The small diameter (<6 mm) of coronary artery and relatively low blood flow provide conditions, under which using synthetic vascular grafts is associated with a high risk of thrombosis, neointima hyperplasia and calcification leading to graft occlusion [7]. Currently, there are no commercially available effective small-diameter vascular prostheses. This makes the development of reliable small-diameter vascular graft extremely important for cardiovascular surgery.

Tissue-engineered vascular grafts are the most promising substitutes for autologous and synthetic grafts. There are various approaches to their development, with the type that mimics the structure and function of the native vascular wall being the most successful [8,9]. This approach allows specialists to maintain an optimal physiological response to the implanted graft, stimulate its integration with surrounding tissues, and reduce the risk of complications after implantation.

The requirements for tissue-engineered vascular graft are as follows: to be biocompatible, to have mechanical properties matching those of the native blood vessels, to stimulate regeneration without causing an inflammatory and immune reaction, to remain non-infected [10]. Moreover, since vascular graft is a blood-contacting device, a high hemocompatibility is another important requirement [11]. The use of synthetic materials, such as biodegradable polyesters, polyester-amides, polyurethanes, for the graft fabrication allows for the control the mechanical properties of the graft [12]. However, synthetic polymers have no cell adhesive sites on their surface, resulting in their low biological activity and biocompatibility [13]. Moreover, the degradation products of synthetic polymers can induce inflammatory and immune responses [14]. Various methods of their modification can be used to increase the biocompatibility of the polymer grafts, for example, coating the surface of the graft with a biocompatible and bioactive material. The surface modification of synthetic graft by biopolymers coating or impregnation promotes the cell adhesion sites formation and imparts biomimetic properties to the material [13]. The most commonly used biopolymers for a vascular graft’s fabrication are collagen, elastin, gelatin and fibrin [7]. Undoubted advantages of fibrin over other biopolymers are its availability and simple and fast isolation from the patient’s blood. The use of this autologous material reduces the risk of transferring infections and immunological reactions. Fibrin fibers contain the sites required for cell adhesion, migration and proliferation, and create an environment that induces tissue formation [15]. In addition, fibrin pre-coating improves cell retention under shear stress, it prevents them from being washed away by a fluid flow [16].

Fibrin is the final product of coagulation cascade [17]. Its polymerization is induced by thrombin and accelerated in the presence of calcium ions. In most research studies, exogenous thrombin from human or animal blood plasma is used to initiate fibrinogen polymerization [18,19]. Despite the measures taken by manufacturers to prevent viral contamination of blood-derived products, there is still a risk of infection transmission [20]. Moreover, for polymerization of fibrin, exogenous thrombin is added to the precipitation solution containing endogenous thrombin, resulting in an excessive thrombin generation and thrombin retention in the fibrin matrix following the completion of polymerization. However, Zhu et al. studied and described the kinetics of thrombin release from clot formed under hemodynamic flow, and the specific conditions necessary for this release [21]. According to the results of the study, the majority (>85%) of generated thrombin was captured by intrathrombus fibrin, however, minimal thrombin fluent in the effluent was still noted. Therefore, there is a certain risk of release of residual exogenous thrombin from fibrin [22]. Exogenous thrombin introduced into the recipient’s body can cause an immune response and production of autoreactive antibodies to the endogenous thrombin, as well as sensibilization and allergic reactions [23,24,25,26]. However, we could not find any published data confirming the presence of thrombin in the fibrin matrix after fibrin polymerization in vitro, and the possibility of its release. Whether the exogenous thrombin is present in the fibrin after its polymerization and whether this fibrin matrix can induce an immune response in vivo remain unclear. Nevertheless, manufacturing completely autologous fibrin is of great interest to researchers. Rieu et al. developed a protocol for the manufacture of fibrin based on thrombin-free gelation pathway of fibrinogen solutions by incubation at 37 °C in mild acidic conditions [27]. The polymerization of fibrinogen under acidic conditions affects the properties of the formed gels. Their main difference from the classic fibrin matrix is instant gelation. Due to such properties, these gels are recommended for bioprinting and electrospinning, where importance lies in high polymerization rates. We have proposed a method for fibrin polymerization by endogenous thrombin. This approach avoids the disadvantages and complications associated with the use of exogenous thrombin and other agents [28].

However, the properties of the final product may differ depending on the method used due to differences in polymerization initiation and the multi-stage cascade of the fibrin polymerization. Therefore, for the further use of autologous fibrin obtained by our proposed method for modifying the surface of vascular grafts, it is important to study its mechanical, structural and biological characteristics, and its effect on hemostasis. In this study, the properties of fibrin matrices fabricated using endogenous thrombin (EnThr fibrin) were investigated and compared with those of fibrin matrices prepared by traditional method in the presence of exogenous thrombin (ExThr fibrin). We found that EnThr fibrin had strength and stiffness more similar to those of the native artery, as well as slower degradation and less thrombogenicity compared to ExThr fibrin. Furthermore, the fabricated fibrin matrices provided high cell adhesion and viability. Hence, the EnThr fibrin matrix might be used for biocompatible coating or impregnation of tissue-engineered vascular grafts and vascular prostheses without the risk of cross-infection.

## 2. Materials and Methods

In this study, peripheral blood, skin biopsies and segments of the internal mammary artery were withdrawn from the patients and healthy volunteers. Written informed consent for the research use of the biological materials was obtained from each participant. The study design received approval by the Ethics Committee of the Research Institute for Complex Issues of Cardiovascular Diseases (protocol number 22, approved 10 December 2015).

### 2.1. Fibrin Formation

#### 2.1.1. Fibrinogen Precipitation

The blood samples were collected into tubes containing 3.8% sodium citrate and centrifuged at 2000× *g* for 10 min. The separated plasma was transferred to clean centrifuge tubes. Precipitate was performed by slowly adding 18% (*v*/*v*) ethanol in HEPES buffer (pH 7.4, 10010031, Gibco, Waltham, MA, USA) to the plasma in a 1:1 ratio with constant stirring. The plasma/ethanol-solution was mixed at 160 rpm using the Mini Rotator/Orbital Shaker (Thermo Fisher Scientific, Waltham, MA, USA) at 0 °C for 1 h then centrifuged at 1200× *g* at 4 °C for 30 min. The supernatant was decanted and precipitate solution was obtained by dissolving precipitate in HEPES-buffer for final fibrinogen concentration in fibrin of 20 mg/mL and 30 mg/mL.

#### 2.1.2. Fibrin Polymerization

Fibrin polymerization was performed in the presence of exogenous thrombin (ExThr fibrin) or endogenous thrombin (EnThr fibrin). In the first case, fibrin matrices were manufactured with the addition of 50 IU/mL exogenous thrombin (T4648, Sigma-Aldrich, Saint Louis, MO, USA) and 0.2% calcium chloride solution (21115, Sigma-Aldrich, Saint Louis, MO, USA) to the precipitate solution. In the second case, the precipitate solution was mixed with 0.2% calcium chloride solution [28]. Next, aprotinin (A1250000, Sigma-Aldrich, Saint Louis, MO, USA) at a final concentration of 100 KIE/mL was added to both solutions to inhibit fibrinolysis. Fibrin polymerization mixtures were immediately poured into molds and allowed to polymerize at room temperature for 1–1.5 h to obtain ExThr or ExThr fibrin samples (Supplementary Figure S1).

### 2.2. Scanning Electron Microscopy (SEM)

ExThr and EnThr fibrin samples were fixed by immersion in 1% glutaraldehyde (G6257, Sigma-Aldrich, Saint Louis, MO, USA) for 24 h, followed by washes with phosphate-buffered saline (PBS, 10010001, Gibco, Waltham, MA, USA) and distilled water. Thereafter, the samples were pre-frozen at −80 °C and then lyophilized in a freeze-dryer Freezone 2.5 (Labconco, Kansas City, MO, USA) at temperature of −40 °C and pressure lower than 0.133 mBar during 2 days.

Samples were mounted on stubs and gold/palladium coating (7 nm) was sputtered on using an EM ACE200 (Leica Mikrosysteme, GmbH, Wetzlar, Germany). Surface structure of samples was investigated with a scanning electron microscope S-3400N (Hitachi, Tokyo, Japan) in the high vacuum mode at an accelerating voltage of 10 kV. Microscope software was used to measure fiber diameter and pore size in each SEM image. At least 50 measurements were conducted for each sample.

### 2.3. Mechanical Testing

Mechanical properties of fibrin matrices were evaluated by uniaxial tensile test in accordance with ISO37:2017. Matrices (*n* = 5 per group) were cut using a custom-shaped knife in the cutting press ZCP 020 (Zwick/Roell, Ulm, Germany). Segments of human internal mammary artery (IMA, *n* = 8), obtained from the patients who underwent coronary artery bypass grafting, were used as controls. Uniaxial tensile tests were performed on the Z-series universal testing machine (Zwick/Roell, Ulm, Germany) using a sensor with a nominal force of 50 N and crosshead speed of 50 mm/min at 37 °C.

The tensile strength was measured by the ultimate stress (MPa). The strain properties of the samples were evaluated by the relative elongation adjusted to the elongation at break (%) and Young’s modulus (MPa) determined in low strains.

### 2.4. Determination of Factor XIII Content

Quantitative measurement of factor XIII (FXIII) in plasma and in the precipitated fibrinogen was performed using Human Factor XIII ELISA Kit (ab108836, Abcam, Cambridge, UK) according to the manufacturer’s instructions. The samples of plasma and 30 mg/mL precipitated fibrinogen without additional dilution were used. The color intensity was measured with Multiskan Sky Microplate Spectrophotometer (Thermo Fisher Scientific, Waltham, MA, USA).

### 2.5. Cross-Linking Analysis of Fibrin by SDS-PAGE

100 μL of either ExThr or EnThr fibrin polymerization mixture containing 20 mg/mL or 30 mg/mL of fibrinogen were poured into Eppendorf tubes. A mixture of 20 mg/mL fibrinogen solution with 50 ME/mL heparin (Belmedpreparaty, Minsk, Belarus) was used as a control. The fibrin samples and controls were diluted in 300 μL of sample buffer containing 0.05 M Tris-HCl (pH = 8.5, Am-0234-0.5, VWR Life Science AMRESCO, Radnor, PA, USA), 8 M urea (Am-0378-0.5, VWR Life Science AMRESCO, Radnor, PA, USA), 2% sodium dodecyl sulfate (SDS, 436143, Aldrich, Saint Louis, MO, USA) and 2% ß-mercaptoethanol (M6250, Sigma-Aldrich, Saint Louis, MO, USA), heated at 100 °C using a Termit solid state thermostat (DNA-Technology, Moscow, Russia) for 20 min followed by the addition of 300 μL of distilled water. 0.1% bromophenol blue (B0126, Sigma-Aldrich, Saint Louis, MO, USA) was used as a tracking dye. Each sample and the protein standard marker Novex Sharp Pre-Stained Protein Standard (LC5800, Thermo Fisher Scientific, Waltham, MA, USA) were loaded into wells (10 μL/well) of NuPAGE 4–12% Polyacrylamide Bis-Tris Gel (1.5 mm thick) (NP0335BOX, Thermo Fisher Scientific, Waltham, MA, USA). Electrophoresis was performed in an XCell SureLock Mini-Cell vertical electrophoresis system (EI0001, Thermo Fisher Scientific, Waltham, MA, USA) using NuPAGE MES SDS running buffer (NP0002, Thermo Fisher Scientific, Waltham, MA, USA) and NuPAGE antioxidant (NP0005, Thermo Fisher Scientific, Waltham, MA, USA) at a voltage of 150 V for 2 h. Gels were stained with 2.5 g/L Coomassie brilliant blue R-250 (B7920, Sigma-Aldrich, Saint Louis, MO, USA) made up in 45% methanol (48638, Vekton, Saint Petersburg, Russia) and 10% acetic acid (127, Vekton, Saint Petersburg, Russia) during 1 h at room temperature on a shaking platform Mini Rotator/Orbital Shaker (Thermo Fisher Scientific, Waltham, MA, USA). The background was removed by boiling gels in 5% acetic acid three times and then shaking overnight with 3% acetic acid at room temperature. The color intensity and size of protein bands were analyzed using ImageJ (U.S. National Institutes of Health; Bethesda, MD, USA). 

### 2.6. Fibrin Degradation

Fibrinolysis and proteolytic degradation of fibrin mediated by plasmin and trypsin were studied in vitro. A total of 0.5 mL of ExThr or EnThr fibrin containing 20 or 30 mg/mL of fibrinogen without aprotinin were polymerized in identical tubes. Then, 1 mL of 0.1 U/mL plasmin (P1867, Sigma-Aldrich, Saint Louis, MO, USA) or 0.1% trypsin (T1426, Sigma-Aldrich, Saint Louis, MO, USA) in 0.9% sodium chloride solution with 0.02% sodium azide (S2002, Sigma-Aldrich, Saint Louis, MO, USA) was gently added to fibrin samples and incubated at 37 °C until complete dissolution. The plasmin and trypsin solutions were changed every 3 days. The size of the fibrin clots was measured every 8 h.

The clot prepared into a cylindrical tube was measured with a ruler and its initial height (h1) was recorded. Then, the height of the clot was measured at certain time points (h2). Degradation (D%) was calculated according to the Equation (1):

D% = (h1 − h2)/h1 × 100%,(1)

### 2.7. Biological Characterization of Fibrin Matrices

Two different cell types, endothelial cells and fibroblasts, were used in these studies in order to characterize the biological properties of fibrin matrices. We chose these cells as endothelial cells form the inner surface of blood vessels, and fibroblasts are highly abundant component in the adventitia, i.e., outer layer of the vascular wall.

#### 2.7.1. Endothelial Cell Culture

To evaluate the cytocompatibility of fibrin matrices, endothelial colony forming cells (ECFCs) at passage 5 were used. ECFCs were isolated from the peripheral blood obtained from healthy volunteers and characterized for morphological features and antigen expression in our own laboratory [29,30]. Cells were cultured in EGM-2MV (CC-3202, Lonza, Basel, Switzerland) supplemented with 5% fetal bovine serum (FBS, 026-100, Cell applications, San Diego, CA USA) into a humidified incubator (Sanyo, Moriguchi, Japan) at 37 °C and 5% CO_2_. After the ECFCs culture reached approximately 70% confluence at passage 5, the cells were dissociated from the culture surface with 0.025% Trypsin/EDTA solution (R001100, Gibco, Waltham, MA, USA). Cells were seeded on ExThr and EnThr fibrin matrices in 24-well plates at a density of 10^4^ cells/well and maintained in culture in complete culture medium for 72 h. Cells cultured without fibrin matrices under similar conditions were used as a control. After 72 h, cell viability was evaluated using both metabolic activity assay and fluorescent dye-based assay.

#### 2.7.2. Metabolic Activity Assay of ECFCs on the Fibrin Surface

A single solution Cell Cytotoxicity Assay Kit-Colorimetric (ab112118, Abcam, Cambridge, UK) based on MTS (5-(3-carboxymethoxyphenyl)-2-(4,5-dimenthylthiazoly)-3-(4-sulfophenyl) tetrazolium) was used. Metabolically active cells reduce tetrazolium salts to formazan products that can be detected colorimetrically [31,32]. The reagent compound (20% of the culture volume) was added to each well containing cells (*n* = 6 per group) and non-cell control wells containing culture medium without cells (*n* = 6). The cells were incubated at 37 °C for 2 h. Then, 200 μL of each sample were transferred to a 96-well plate, and optical density (OD) was measured at 570 nm and 605 nm using a Multiskan Sky Microplate Spectrophotometer (Thermo Fisher Scientific, Waltham, MA, USA).

The metabolic activity of cells was calculated according to the Equation (2):

Metabolic activity = R_sample_ − R_0_, (2)

where, R_sample_ is the absorbance ratio of OD_570_/OD_605_ in the presence of the reagent compound; R_0_ is the averaged background absorbance ratio of OD_570_/OD_605_ for non-cells control well containing culture medium and reagent compound without cells.

#### 2.7.3. Fluorescent Dye-Based Viability Assay of ECFCs on the Fibrin Surface

Cell-populated fibrin matrices and control cells cultured on plastic (*n* = 3 per group) were stained with 10 μg/mL Hoechst 33342 (14533, Sigma-Aldrich, Saint Louis, MO, USA) for 10 min. To identify dead cells, additional staining with 30 μg/mL ethidium bromide (AppliChem, Darmstadt, Germany) was performed for 1 min. The number of cells on the fibrin samples and culture plastic was assessed using the inverted fluorescence microscope Axio Observer Z1 (Carl Zeiss, Oberkochen, Germany), and 10 random fields of view per sample were analyzed. Next, the number of cells counted in the fields of view was converted to cells per 1 mm^2^ (Equation (3)):

Number of cells (cells/mm^2^) = Number of cells in the field of view/Area of the field of view (mm^2^). (3)

The number of living cells was calculated by subtracting the number of dead cells from the total number of cells.

Cell viability was defined as (Equation (4)):

Cell viability (%) = Number of living cells × 100/Total number of cells.(4)

#### 2.7.4. ECFC Focal Adhesion on the Fibrin Surface

To evaluate cellular adhesion, the focal adhesion protein paxillin was detected. For this purpose, three Well Chambers (80381, Ibidi, Graefelfing, Germany) were coated with either ExThr or EnThr fibrin (*n* = 3 per group). ECFCs were seeded at the density of 2 × 10^4^ cells/well and cultured in complete medium for 72 h. Cells cultured in three Well Chambers without fibrin matrices were used as a control (*n* = 3). Subsequently, all samples were fixed in 4% paraformaldehyde (J61899, Alfa Aesar, Haverhill, MA, USA) for 10 min and then permeabilized with 0.1% Triton X-100 (X100, Sigma Aldrich, Saint Louis, MO, USA) for 15 min. To prevent the nonspecific binding of antibodies, PBS containing 1% bovine serum albumin (BSA, A9647, Sigma-Aldrich, Saint Louis, MO, USA) was added to each well and the samples were incubated at room temperature for 1 h. Incubation with primary Recombinant Anti-Paxillin antibodies (ab32084, Abcam, Cambridge, UK) at dilution of 1:200 was performed at 4 °C overnight. Samples were washed by wash buffer (PBS buffer with 0.1% Triton X-100) and then incubated for 1.5 h with Donkey anti-Rabbit IgG (H + L) Highly Cross-Adsorbed Secondary Antibody Alexa Fluor 488 (A21206, Thermo Fisher, Waltham, MA, USA) at dilution of 1:600 and Phalloidin Alexa Fluor 568 (A12380, Invitrogen, Waltham, MA, USA) at dilution of 1:40. Samples were rinsed with wash buffer to remove unbound secondary antibodies and cell nuclei were counterstained with 10 μg/mL DAPI (D9542, Sigma-Aldrich, Saint Louis, MO, USA) for 40 min. Finally, slides were mounted under coverslips in ProLong reagent (P36930, Life Technologies, Carlsbad, CA, USA) and viewed using a confocal scanning microscope LSM 700 (Carl Zeiss, Oberkochen, Germany).

#### 2.7.5. Fibroblast Cell Culture

Dermal fibroblasts were isolated from human skin biopsies according to the protocol of the University of Helsinki «Fibroblast cultures from skin biopsy» [33]. The obtained cell culture exhibited morphology similar to human fibroblasts and the phenotype CD90^+^CD73^+^CD45^−^CD31^−^. Fibroblasts were cultured in complete DMEM (31885023, Gibco, Waltham, MA, USA) supplemented with 10% FBS, 100 U/mL of penicillin and 100 μg/mL of streptomycin (10378016, Gibco, Waltham, MA, USA), 2 mM L-glutamine (A291680, Gibco, Waltham, MA, USA), 0.25 μg/mL amphotericin B (15290026, Gibco, Waltham, MA, USA) and 20 mM HEPES (pH 7.4–7.5). For the experiment, cells were used from passage 3.

#### 2.7.6. Viability Assay of Fibroblasts within Fibrin Matrices

Both ExThr and EnThr fibrin polymerization mixtures were carefully mixed with fibroblast at final concentration of 10^5^ cell per 1 mL of fibrin and then added to 24-well culture plates (1 mL/well, *n* = 4 per group). The thickness of the fibrin matrices was 5 mm. Cell-containing samples of ExThr and EnThr fibrin were cultured in complete DMEM for 14 days. The medium was changed every 72 h. After 14 days, the fibrin matrices were carefully detached from the wells and fibroblast viability was determined as described for ECFCs in 2.7.3. Cells were visualized separately on the upper surface (top) and on the reverse side of fibrin (bottom).

### 2.8. Assessment of Thrombogenicity of Fibrin Matrices

#### 2.8.1. Platelet Aggregation

To evaluate platelet aggregation, fresh donor blood was collected into the tubes containing sodium citrate at a ratio of 1:9 (citrate: blood). Platelet-rich plasma (PRP) was obtained by centrifugation of the blood at 150 g for 7 min. ExThr fibrin (*n* = 6) and EnThr fibrin (*n* = 6) matrices were exposed to PRP at 37 °C for 5 min. Intact PRP was used as a control (*n* = 6). Spontaneous and induced platelet aggregation by 4 μm/L adenosine 5’-diphosphate (030, Tehnologia-standart, Barnaul, Russia) were measured using APACT 4004 platelet analyzer (LABiTec, Ahrensburg, Germany). 

#### 2.8.2. Blood Coagulation

We used the test of activated partial thromboplastin time (APTT) for assessing fibrin contact activation of coagulation pathway. Platelet-poor plasma (PPP) was obtained by centrifuged citrate blood at 2000× *g* for 15 min. EnThr (*n* = 7) and ExThr (*n* = 7) fibrin samples (5 × 5 × 3 mm) were placed in 24-well plate and incubated with 2 mL PPP on the Mini Rotator/Orbital Shaker at 100 rpm at 37 °C for 30 min. Intact PPP was used as a control (*n* = 7). APTT assay was performed on Sysmex CA-500 coagulation analyzer (Sysmex, Kobe, Japan) employing APTT reagent Pathromtin SL (Siemens Healthcare Diagnostics Products GmbH, Munich, Germany).

### 2.9. Thrombin Generation Assay (TGA)

Thrombin generation was evaluated in the liquid obtained after retraction of fibrin matrices (2 h after pouring in the matrix for polymerization). Prepared fibrin matrices were washed three times with 0.9% sodium chloride solution for 24 h. Then, fibrin samples were incubated in 0.9% sodium chloride solution for 8 h, and the presence of thrombin in this solution was also studied. 

The analysis was performed on the Ceveron alpha automatic analyzer with a TGA module (Technoclone, Vienna, Austria) using a TGA RC Low Kit (5006013, Technoclone, Vienna, Austria) and dedicated control plasmas (low and high). A sample (liquid after retraction of fibrin clots or after washing in 0.9% sodium chloride solution) and an activator were added to the tube. The mixture was incubated at 37 °C followed by the addition of a buffer containing ionized calcium and a fluorogenic substrate. Thrombin cleaved the substrate, resulting in the release of a fluorophore molecule, which was automatically recorded by the fluorometer at regular intervals. The luminescence intensity was proportional to the rate of fluorescence change and, consequently, to the thrombin concentration.

The Ceveron alpha TGA software automatically calculated the following results: lag time from the time point when the TGA reagent including CaCl_2_ added in until the first burst in thrombin formation (min); peak thrombin, i.e., maximum of thrombin concentration (nM/L); area under the curve (AUC, nM).

### 2.10. Statistical Analysis

For statistical analysis, GraphPad Prism software version 7 (GraphPad Software, San Diego, CA, USA) was used. The obtained data were expressed as the median and interquartile range (25th and 75th percentiles). Differences between the independent groups were assessed using the Mann–Whitney U-test. Three or more independent groups were compared using the Kruskal–Wallis one-way ANOVA test with a false discovery rate (FDR) correction for multiple testing. Statistical *p*-values < 0.05 were considered to be statistically significant.

## 3. Results

### 3.1. Microstructure of Fibrin Matrices

**Figure 1:**
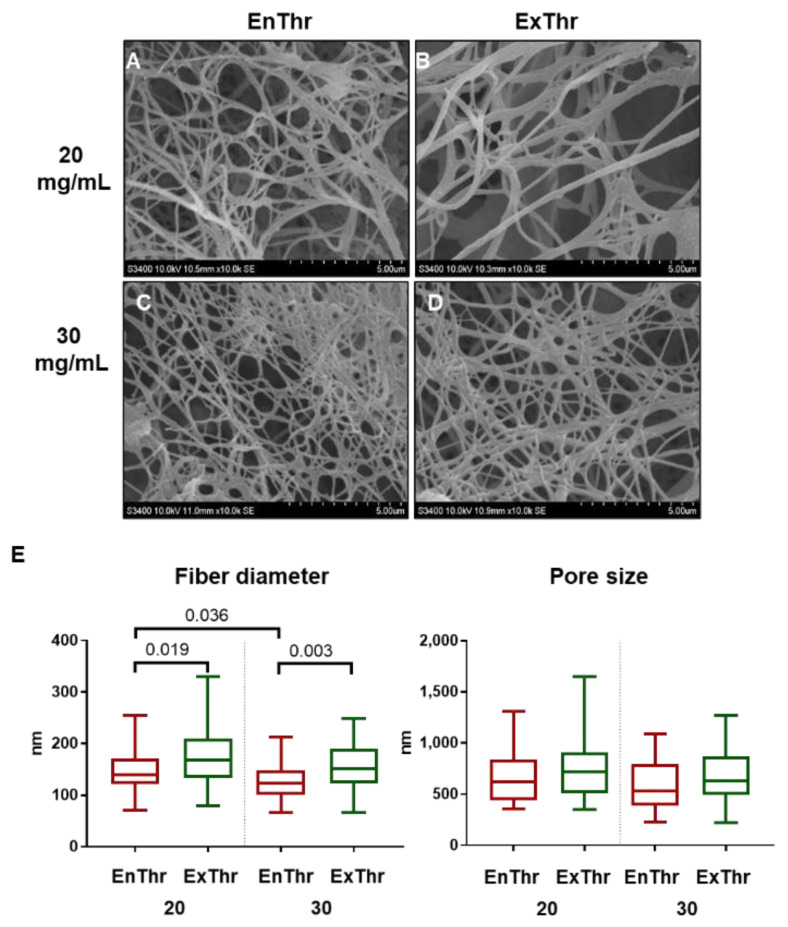
Microstructure of fibrin matrices prepared at fibrinogen concentrations of 20 and 30 mg/mL using endogenous (EnThr) and exogenous (ExThr) thrombin: (**A**–**D**) scanning electron microscopy of fibrin matrix surface, ×10,000 magnification; (**E**) quantification analysis of fiber diameter and pore size, center lines indicate the median, box boundaries indicate the 25th and 75th percentiles and whiskers indicate range.

The SEM images of all fibrin samples exhibited fibrous structure (Figure 1A–D). The fibrin network of EnThr containing 20 mg/mL fibrinogen (EnThr 20) consisted of thinner fibers than the ExThr fibrin samples with 20 mg/mL fibrinogen (ExThr 20) (*p* < 0.05, Figure 1A,B,E). Furthermore, EnThr fibrin matrix containing 30 mg/mL fibrinogen (EnThr 30) was composed of thinner fibers compared to EnThr 20 (*p* < 0.05, Figure 1A,C,E). No differences in pore size were found between the ExThr and EnThr fibrin samples.

### 3.2. Mechanical Properties of Fibrin Matrices

**Figure 2:**
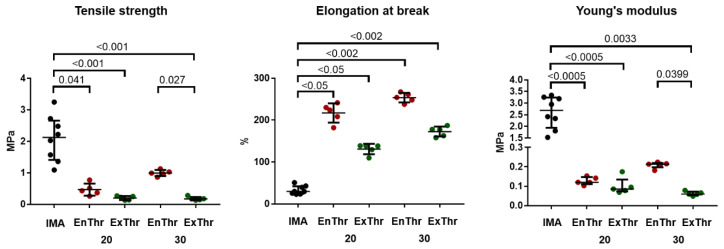
Mechanical properties of the fibrin matrices prepared at fibrinogen concentrations of 20 and 30 mg/mL using endogenous (EnThr) and exogenous (ExThr) thrombin as compared to the internal mammary artery (IMA). Center lines indicate the median, whiskers indicate the 25th and 75th percentiles.

The mechanical properties of four groups of fibrin matrices were estimated. Segments of the IMA- were used as a control, since the mechanical properties of vascular grafts should be most similar to those of native vessels. As shown in Figure 2, tensile strength of ExThr 20 (0.22 (0.14–0.26) MPa), ExThr 30 (0.17 (0.14–0.23) MPa) and EnThr 20 (0.43 (0.32–0.64) MPa) samples was lower compared to IMA (2.26 (1.29–2.85) MPa) (*p* < 0.05), whereas tensile strength of EnThr 30 fibrin matrix exceeded that of ExThr 30 by 5.5-fold (*p* < 0.05). Young’s modulus of EnThr 20 and all types of ExThr samples were lower than in IMA. Likewise, Young’s modulus of EnThr 30 matrix (0.21 (0.19–0.22) MPa) was 3.5 times higher than ExThr 30 samples (0.06 (0.04–0.07) MPa) (*p* < 0.05). Moreover, tensile strength and Young’s modulus of EnThr 30 did not differ significantly from those of IMA. In addition, elongation at break of fibrin matrices exceeded that of IMA (*p* < 0.05).

### 3.3. Content of FXIII in Fibrinogen Precipitate

Blood plasma used for the fibrinogen precipitation contained 22.20 (21.35–23.05) μg/mL FXIII. The concentration of FXIII in the precipitate solution containing 30 mg/mL fibrinogen was 106.50 (99.25–109.4) μg/mL. To obtain 1 mL of 30 mg/mL fibrinogen, 5 mL of plasma were required. The calculation of the relative yield of FXIII showed that the 93.77 (93.02–95.9)% of the initial concentration of FXIII in plasma was retained in the precipitate. 

### 3.4. Detection of α-α and γ-γ Cross-Links by SDS-PAGE

**Figure 3:**
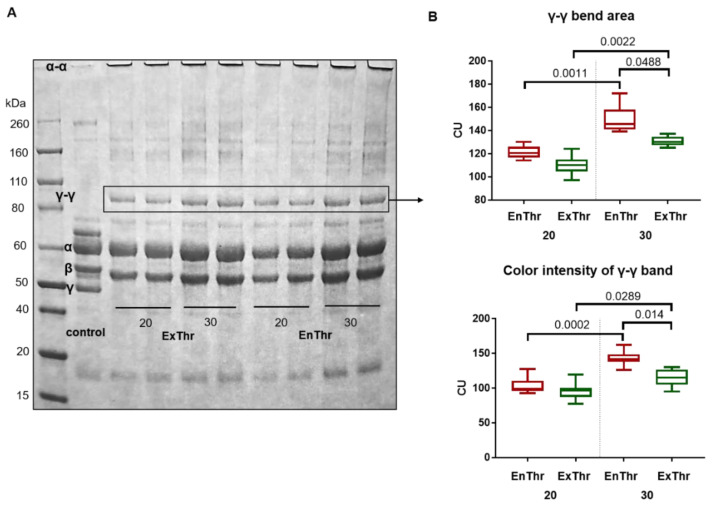
Cross-linking of fibrin matrices prepared at fibrinogen concentrations of 20 and 30 mg/mL using endogenous (EnThr) and exogenous (ExThr) thrombin: (**A**) SDS-PAGE analysis of fibrin matrices as compared to the unpolymerized fibrin solution (control); (**B**) quantitative analysis of γ-γ dimer bands, center lines indicate the median, box boundaries indicate the 25th and 75th percentiles and whiskers indicate range.

We performed SDS-PAGE to identify of α-α and γ-γ cross-links in both EnThr and ExThr fibrin samples. In the control sample containing 20 mg/mL fibrinogen solution, monomers of the Aα-, Bβ- and γ-chains were determined. The presence of A and B fibrinopeptides in the α- and β-chains of the control is evident from a slight shift of their band towards smaller molecular mass in fibrin samples. In the EnThr and ExThr fibrin samples, γ-chain monomers were absent, but γ-γ dimers formation was detected (Figure 3A).

Quantitative analysis of the bands showed bigger size and more color intensity in the γ-γ dimer band of EnThr 30 fibrin compared to ExThr 30 fibrin (Figure 3B). In turn, a greater amount of γ-γ cross-links in the EnThr fibrin compared to ExThr fibrin, with an equal fibrinogen concentration in the samples (20 or 30 mg/mL), indicates a higher FXIII activity in EnThr fibrin. Since cross-linked α-α chains are very high molecular weight oligomers, they were localized in the area of the gel wells.

### 3.5. Degradation of Fibrin Matrices

**Figure 4:**
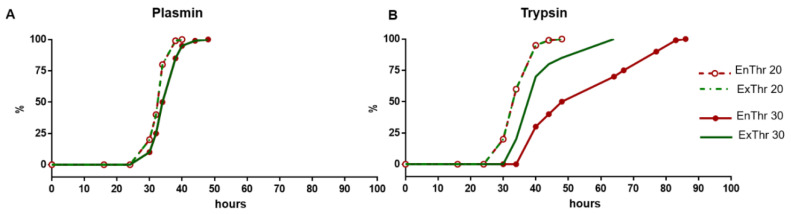
Degradation rate of fibrin matrices prepared at fibrinogen concentrations of 20 and 30 mg/mL using endogenous (EnThr) and exogenous (ExThr) thrombin: (**A**) fibrin degradation by 0.1 U/ml plasmin; (**B**) fibrin degradation by 0.1% trypsin.

Cross-links of fibrin fibers increase both the mechanical strength of the clot and resistance to degradation by fibrinolytic and proteolytic enzymes [34,35]. We measured the degradation rate of fibrin samples without aprotinin. Fibrinolysis of fibrin was stimulated by plasmin (Figure 4A), and proteolytic degradation was carried out using trypsin (Figure 4B). 

Both plasmin- and trypsin-induced degradation of fibrin with a higher fibrinogen content (30 mg/mL) was slower than that of fibrin with lower fibrinogen concentration (20 mg/mL) (Figure 4A,B). This may be related to higher density of fibrin clots with 30 mg/mL fibrinogen. Plasmin-induced degradation of matrices with 20 and 30 mg/mL fibrinogen was completed after 40 and 48 h, respectively (Figure 4A). We did not find any differences in the rate of fibrinolysis between samples EnThr and ExThr containing an equal concentration of fibrinogen.

The study of the proteolytic degradation of fibrin matrices showed that EnThr 20 and ExThr 20 were completely degraded by trypsin after 48 h, and the time of trypsin-induced degradation of EnThr 30 and ExThr 30 was 64 and 84 h, respectively (Figure 4B). Consequently, the EnThr 30 samples demonstrated a greater resistance to proteolytic degradation than ExThr 30. No differences were found in the rate of proteolytic degradation between EnThr 20 and ExThra 20 fibrin matrices.

### 3.6. Biological Properties

The biological properties of fibrin matrix did not differ between the groups containing 20 and 30 mg/mL fibrinogen (Supplementary Figure S2). Therefore, we presented the results of testing EnThr 30 and ExThr 30 fibrin matrices as more promising for tissue engineering.

#### 3.6.1. Cytocompatibility of Fibrin Matrices

**Figure 5:**
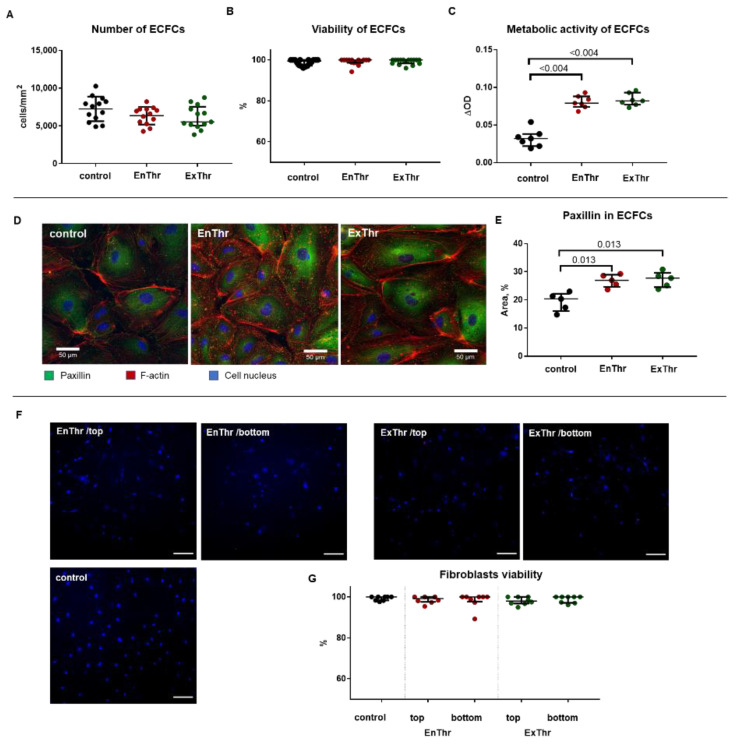
Biological properties of fibrin matrices prepared using endogenous (EnThr) and exogenous (ExThr) thrombin: (**A**) number of the endothelial cells on the fibrin surface; (**B**) viability and (**C**) metabolic activity of the endothelial colony forming cells (ECFCs); (**D**) immunofluorescent staining of focal adhesions in endothelial cell (paxillin-green, F-actin–red, cell nucleus-blue), scale bar 50 μm, ×400 magnification; (**E**) quantitative analysis of paxillin; (**F**) fluorescent analysis of fibroblast viability on the top and bottom sides of the fibrin matrices (dead cells–red, cell nucleus–blue), scale bar 100 μm, ×100 magnification; (**G**) quantitative analysis of fibroblast viability. Center lines indicate the median, whiskers indicate the 25th and 75th percentiles.

We evaluated the cytocompatibility of ExThr and EnThr fibrin matrices using endothelial colony-forming cells (ECFCs). ECFCs were seeded on the fibrin surface, and, the number of cells, their viability, metabolic activity and adhesion were assessed. Similar high cell viability (94–100%) was observed after 3 days of cell culturing on the surface of ExThr and EnThr fibrin and in control (cell culture plastic) (Figure 5B).

The results showed no difference in cell metabolism in both fibrin matrices types (Figure 5C). It is worth noting that the metabolism of endothelial cells cultured on EnThr and ExThr matrices was approximately 2.5 times higher than that of cells incubated on the surface of the culture plastic (Figure 5C). To exclude the possibility of a decrease in cell metabolism due to cell overgrowth, we performed a cell count. The cell number did not differ significantly among EnThr and ExThr matrices and control (cell culture plastic) (Figure 5A). 

Cell adhesion is another important characteristic that determines the viability of cells on the surface of a biomaterial. Cell adhesion to biomaterial depends on the ability of the substrate to activate the integrin receptors and focal adhesions formation [36]. We determined the area of focal adhesion of endothelial cells by staining the scaffold protein paxillin, which provides the binding of integrin to the cytoskeleton and regulates the focal adhesions assembly [37]. Cell area occupied by paxillin in cells cultured on the surface of ExThr fibrin did not differ from that in cells on EnThr samples (Figure 5D,E), while the area of focal adhesion in cells cultured on both types of fibrin matrices was 1.3-fold larger than that in control.

#### 3.6.2. Viability of Fibroblasts Incorporated into Fibrin Matrices

Next, we studied the long-term viability of fibroblast incorporated into fibrin matrices to evaluate the ability of fibrin matrix to supply the cells with oxygen and nutrients and remove waste products. We seeded human fibroblasts in ExThr and EnThr fibrin matrices and allowed the cells to grow for 14 days. Results showed that cell viability was 89–100% with no differences among ExThr and EnThr fibrin samples and control (Figure 5F,G). Moreover, there were no differences in cell viability between fibroblasts located on the top and bottom (5 mm deep) of the fibrin matrices. 

### 3.7. Thrombogenicity of Fibrin Matrices

Lastly, we assessed the influence of fibrin matrices on the intrinsic coagulation pathway and platelet aggregation.

**Figure 6:**
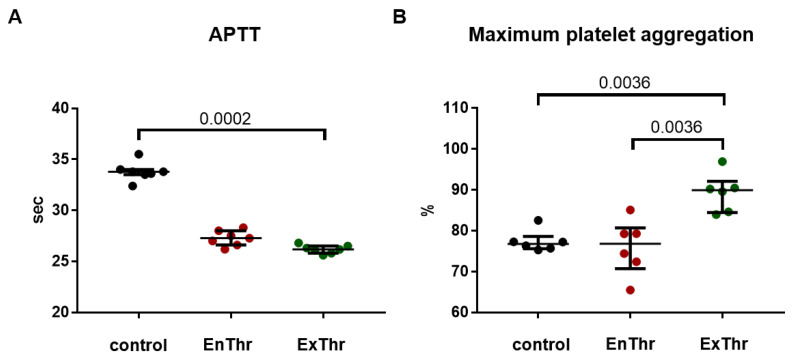
Thrombogenicity of fibrin matrices prepared using endogenous (EnThr) and exogenous (ExThr) thrombin: (**A**) activated partial thromboplastin time (APTT) and (**B**) maximum platelet aggregation. Center lines indicate the median, whiskers indicate the 25th and 75th percentiles.

As shown in Figure 6A, the APTT were reduced by EnThr (27.27 (26.6–28.0) sec) and ExThr fibrin (26.18 (25.8–26.8) sec) compared with that of control (33.8 (33.5–35.5) sec), which indicated a significant activation of the intrinsic coagulation pathway by both fibrin matrices. ExThr fibrin exhibited the most pronounced maximum platelet aggregation, which was 1.12-fold higher than that of EnThr fibrin and 1.17-fold to control (Figure 6B). These data suggest that EnThr fibrin matrix showed less thrombogenicy compared to ExThr fibrin. 

### 3.8. Residual Thrombin Present in Fibrin Matrices

A trace amount (20.6 nM/L) of thrombin was found in the ExThr 20 fibrin before washing, however it was not detected in EnThr 20, ExThr 30, EnThr 30 samples (Table 1, Supplementary Figure S3). After thorough washing of the ExThr 20 fibrin matrix with 0.9% sodium chloride solution, no release of residual thrombin from fibrin was noted.

## 4. Discussion

Synthesis of completely autological fibrin attracts great interest due to their potential applications in biomedical fields. No need for traditional administration of exogenous thrombin reduces the cost and risks of cross-infection since clinically used thrombin is not autologous [27]. We have proposed a method for producing fibrin without the use of exogenous thrombin, which may overcome the above-mentioned disadvantages. However, the proposed method of fibrin polymerization can affect the properties of the fibrin matrix. In this study, we compared the biological, mechanical and structural properties of fibrin matrices prepared using endogenous and exogenous thrombin.

Autologous fibrinogen was isolated from blood plasma by ethanol precipitation using low concentration of ethanol. The ethanol precipitation provides a maximum yield of fibrinogen (up to 80% of its total plasma content) for a minimum period of time, and it does not adversely affect the mechanical and biological properties of fibrin synthesized from this fibrinogen [38,39]. Fibrinogen concentrations were chosen according to the literature data and the data obtained in our previous study that indicate that high fibrinogen content (10–30 mg/mL) is most suitable for the fabrication of cardiovascular tissue-engineered implants [18,40,41].

The biological characterization of fibrin matrices was performed with endothelial cells and fibroblasts using different methods for determining cell viability. Fluorescent dye-based method used in this study detects presence of dead cells. Colorimetric tetrazolium salt assay helps to measure redox activity in metabolically active cells. Metabolism can be considered a more sensitive indicator than cell death, since the decline in cell metabolic activity occurs earlier than their death [32]. Experiments showed that the cytocompatibility of fibrin matrices prepared with endogenous thrombin did not differ from that of the samples prepared with exogenous thrombin. Cells seeded on all types of fibrin samples demonstrated high cellular viability, metabolic activity and adhesion. Moreover, the results indicated that fibrin matrices with incorporated cells can potentially be used for the fabrication of the middle and/or outer layers of tissue-engineered vascular grafts [42].

We found that mechanical properties of ExThr and EnThr fibrin matrices were weaker than those of internal mammary artery. This limits the use of fibrin as a base material for fabrication of vascular grafts. Therefore, the fibrin obtained by our method could be used for coating or impregnation of tissue-engineered vascular grafts and vascular prostheses.

Significant differences in such related characteristics as structure, strength, and elastic properties were found between different types of fibrin matrices. SEM imaging of the matrices revealed finer fibers in the structure of EnThr fibrin with a fibrinogen concentration of 30 mg/mL compared to ExThr fibrin with equal fibrin content. Moreover, EnThr samples displayed tensile strength and stiffness higher than those of the ExThr fibrin. Thus, we have noted that fibrin matrix with small diameter fibers and decreased pore size possessed higher strength and stiffness. This also was observed and confirmed and explained by other authors. Li et al. showed that the stiffness of fibrin fibers is inversely proportional to their diameters [43]. This can be explained by the non-uniform packing of protofibrils into fibrin fibers. Thinner fiber has greater stiffness and strength than thick fiber due to a higher packing density of protofibrils. However, FXIII-mediated cross-linking plays an important role in compacting and maturation of fibrin fibers [44].

The mechanical properties of fibrin clots are highly dependent on the number of fibrin fiber cross-links. Under physiological conditions cross-linking of fibrin fibers is catalyzed by the activated factor XIII (FXIII), a member of the transglutaminase family. FXIII-induced cross-linking has a significant effect on the mechanical properties of the fibrin clot. Several studies have found a 2–5-fold increase in clot stiffness after cross-linking by FXIII [45,46]. Activated FXIII catalyzes the formation of intermolecular ε-lysyl-γ-glutamyl covalent bonds between the C-terminal domains of the fibrin γ- and α-chains. [47,48].

An increase in fibrin fiber density in the presence of FXIII has been demonstrated by Hethershaw et al. [49]. Cross-linking of fibers leads to the formation of more rigid clots without pronounced changes in the structure of the fibrin network. Fiber compaction occurs due to the formation of cross-links between the protofibrils within fibrin fiber. Maturation of fibers results from the lateral aggregation of protofibrils and FXIII-mediated cross-linking between the α-chains and γ-chains. The fibrin fibers become tightly packed, thin, strong and rigid. Furthermore, cross-linking by FXIII reduces pore size in the clot [49].

We found that a significant part of FXIII remained in the precipitate isolated by the ethanol precipitation technique at a low ethanol concentration. EnThr and ExThr fibrin samples contained the equal amount of FXIII. However, the activation of the FXIII was carried out by two distinct methods. In the case of EnThr fibrin, FXIII was activated only in the presence of endogenous thrombin, while in ExThr fibrin, both endogenous and exogenous thrombin were used. Differences in both types of thrombin were present and thrombin concentration could influence the FXIII activity. FXIII activity can be characterized by the number of specific cross-links between γ-γ and α-α chains in the EnThr and ExThr fibrin samples. FXIII-induced cross-linking of the fibrin starts with the formation of covalent bonds between γ-chains and then cross-linking occurs between α-chains. The formation of hybrid α-γ cross-links in fibrin clots is also described [32,50].

It is also known that γ-γ cross-linking contributes more to the increase in the viscoelastic properties of the fibrin network [48,51]. The formation of γ-γ cross-links provides the production of oligomers that contribute to the increased stiffness and strength of the clot [32]. Our findings are consistent with these data [4,51] and reveal a greater amount of γ-γ dimers in the structure of EnThr fibrin, which had a higher strength and stiffness than ExThr fibrin.

FXIII also has a significant effect on fibrin degradation. FXIII-mediated cross-linking of fibrin increases its resistance to fibrinolysis. On the one hand, this is due to the cross-linking of α2-antiplasmin and the α-chain of fibrin by FXIIIa [52] that prevents the displacement of the plasmin inhibitor from the fibrin clot during retraction [34]. On the other hand, cross-linking of protofibrils and fibrin fibers directly slows down the degradation process [50]. Fibrin networks cross-linked by FXIII consist of thinner and tighter fibers and smaller pores, which make them more resistant to fibrinolysis compared to non-crosslinked networks of thicker fibers and larger pores [53]. In addition, thin and tightly packed fibrin fibers exhibit reduced binding of tissue plasminogen activator and plasminogen resulting in a decrease in the rate of plasminogen conversion to plasmin [54,55]. However, these structural changes have a less significant effect on fibrinolysis compared to retraction or mechanical compaction of fibrin clots in the presence of FXIII. Retraction and compaction bring the fibrin fibers into close contact, predisposing them to additional crosslinking by FXIIIa and the incorporation of α2-antiplasmin into the fiber structure. Rijken et al. showed that lysis of the compacted clots was strongly inhibited by FXIIIa as it prevented the a2-antiplasmin displacement from the clot during retraction. 31.2% of a2-antiplasmin remained bound to the clot in the presence of FXIIIa, whereas in the absence of the factor, only 3.9% of bound inhibitor was noted [34]. Another mechanism that reduces the degradation of fibrin during retraction and compaction, is associated with a decrease in clot permeability and in the diffusion of fibrinolytic proteins [49,56].

Structural features of the fibrin network and fiber lysis profile of fibrin without retraction are difficult to detect using standard approaches to studying fibrinolysis [34]. Studying fibrinolysis using the Chandler loop method always involves mechanical compaction of fibrin [34,57]. In contrast, we assessed the rate of fibrin degradation without compaction of clots, which remained attached to the walls of the tube throughout the experiment. Formation of α-α cross-links and γ-dimers occurs at an early stage of the clot-forming process and does not significantly increase the fibrinolysis time [34]. For these reasons, we did not find any significant differences in fibrinolytic activity between samples prepared by different polymerization methods. However, a decrease in the fibrinolysis rate of matrices with a high content of fibrinogen (30 mg/mL) was noted, and supposedly, it occurred due to fibers being much thinner in these samples compared to fibers of fibrins containing 20 mg/mL fibrinogen The stability of the fibrin clot was clearly demonstrated in the proteolytic degradation experiment using trypsin. The proteolytic degradation of EnThr fibrin with 30 mg/mL fibrinogen was slower than that of the ExThr fibrin containing equal concentration of the fibrinogen.

Our results indicate a higher efficiency of the method in simulating fibrin polymerization using autologous endogenous thrombin, which provides the formation of a larger number of γ-γ cross-links, and, consequently, fiber compaction and improved resistance to degradation. We assume that a less efficient polymerization and activation of FXIII in ExThr samples, which is most noticeable at high fibrinogen concentrations (30 mg/mL) in fibrin, may result in competitive partial displacement of more specific endogenous thrombin by less specific exogenous thrombin, whereas, only the more effective endogenous thrombin is involved in the polymerization of EnThr fibrin. In the case of ExThr fibrin, polymerization is carried out in the presence of both exogenous and autologous endogenous thrombin, which probably compete with each other for binding to fibrinogen.

The low thrombogenicity of the material is extremely important for the vascular grafts, since it determines the graft’s patency. EnThr fibrin matrix showed less thrombogenicity in vitro, as it caused less platelet aggregation than ExThr fibrin. However, further study of the causes and mechanisms of lower platelet activation by fibrin matrices prepared without the use of exogenous thrombin is required. We did not find significant differences in the activation of coagulation hemostasis between ExThr and EnThr fibrin matrices that is consistent with our results showing no thrombin release from the washed matrices. 

In this study, an in vitro assessment of the proposed method of fibrin polymerization was carried out. This work is a first step in the evaluation of feasibility of using this method for the manufacture of fibrin coating for vascular tissue engineering before in vivo studies.

## 5. Conclusions

The proposed method for fibrin polymerization without the use of exogenous thrombin allows us to produce a completely autologous fibrin matrix with high biocompatibility. The use of this matrix for the coating and impregnation of vascular implants reduces the risk of cross-infection. Moreover, the obtained fibrin has a high cytocompatibility, optimal strength and stiffness, increased resistance to degradation and reduced thrombogenicity. Thus, this method could be an alternative to the typical production method of fibrin for the surface modification of personalized vascular grafts and other medical devices in contact with blood.

## Figures and Tables

**Table 1:** Thrombin generation assay of the fibrin matrices before and after washing.

Sample	Lag Time, min	Peak Thrombin, nM/L	AUC, nM
High control plasms	2.4	190.1	3040.3
Low control plasma	4.0	120.1	2261.2
ExThr 20 before washing	0.5	20.6	847.2
ExThr 30 before washing	-	-	-
EnThr 20 before washing	-	-	-
EnThr 30 before washing	-	-	-
ExThr 20 after washing	-	-	-

ExThr 20—fibrin matrix prepared at 20 mg/mL fibrinogen using exogenous thrombin; EnThr 20—fibrin matrix prepared at 20 mg/mL fibrinogen using endogenous thrombin; ExThr 30—fibrin matrix prepared at 30 mg/mL fibrinogen using exogenous thrombin; EnThr 30—fibrin matrix prepared at 30 mg/mL fibrinogen using endogenous thrombin; AUC—area under the curve

## Data Availability

The data presented in this study are available on request from the corresponding author.

## Supplementary Materials

The following supporting information can be downloaded at: https://www.mdpi.com/article/10.3390/biomedicines10040789/s1, Figure S1: Fibrin matrices prepared at fibrinogen concentrations of 20 and 30 mg/mL using endogenous (EnThr) and exogenous (ExThr) thrombin; Figure S2: Biological properties of fibrin matrices prepared at fibrinogen concentrations of 20 and 30 mg/mL using endogenous (EnThr) and exogenous (ExThr) thrombin; Figure S3: Thrombin generation assay (TGA) profile.
