# Supplementary material for: Advantages of Fibrin Polymerization Method without the Use of Exogenous Thrombin for Vascular Tissue Engineering Applications
Source: Biomedicines. 2022 Mar 28;10(4):789. doi: 10.3390/biomedicines10040789 (PMC9026760; doi:10.3390/biomedicines10040789)
Supplement: Supplementary file 1 [file biomedicines-10-00789-s001.zip › biomedicines-1619938-supplementary.pdf]

Supplementary Figure S1

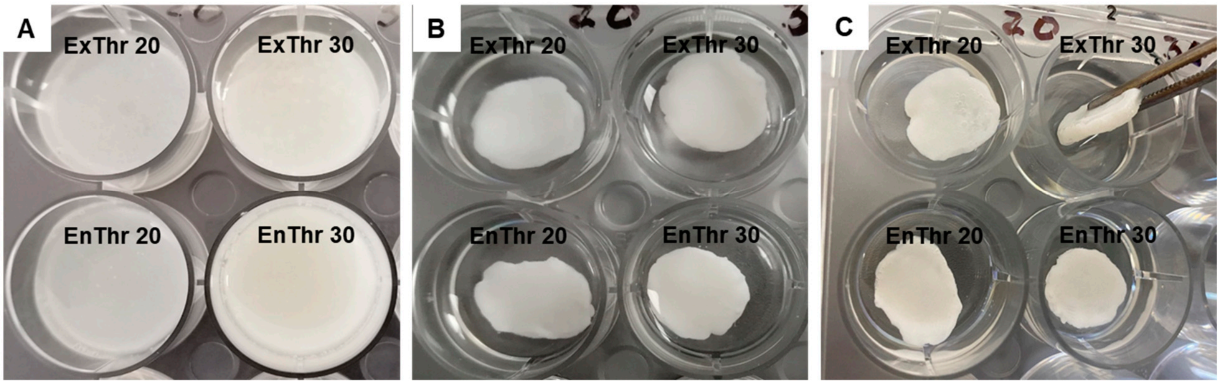

Supplementary Figure S1. Fibrin matrices prepared at fibrinogen concentrations of 20 and 30 mg/mL using endogenous (EnThr) and exogenous (ExThr) thrombin: (A) 1 h of fibrin polymerization; (B-C) fibrin retraction.

Supplementary Figure S2

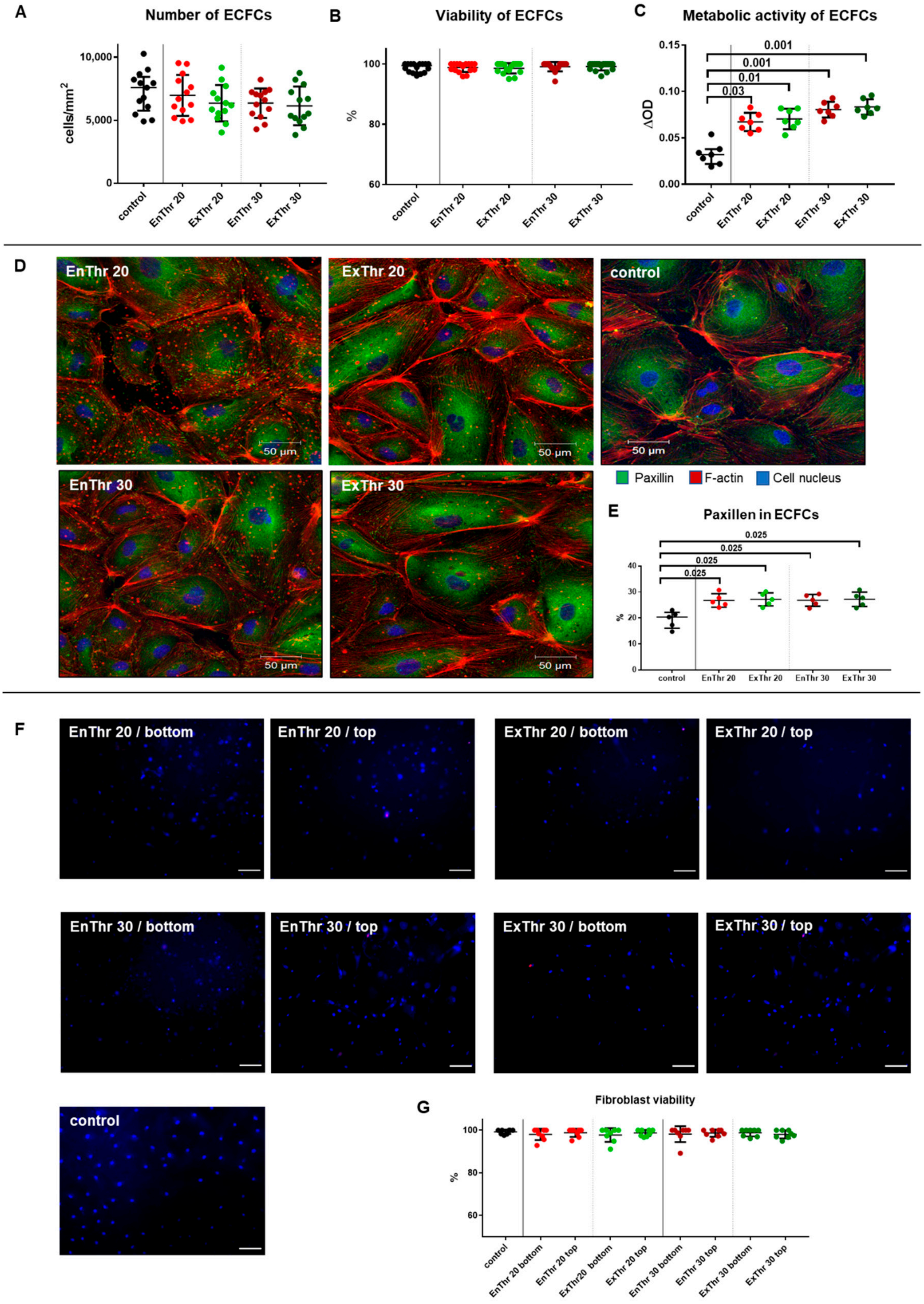

Supplementary Figure S2. Biological properties of fibrin matrices prepared at fibrinogen concentrations of 20 and 30 mg/mL using endogenous (EnThr) and exogenous (ExThr) thrombin: (A) number of the endothelial cells on the fibrin surface; (B) viability and (C) metabolic activity of the endothelial colony forming cells (ECFCs); (D) immunofluorescent staining of focal adhesions in endothelial cell (paxillin - green, F-actin – red, cell nucleus - blue), scale bar 50 µm, ×400 magnification; (E) quantitative analysis of paxillin; (F) fluorescent analysis of fibroblast viability on the top and bottom sides of the fibrin matrices (dead cells – red, cell nucleus – blue), scale bar 100 µm, ×100 magnification; (G) and quantitative analysis of fibroblast viability. Center lines indicate the median, whiskers indicate the 25th and 75th percentiles.

Supplementary Figure S3

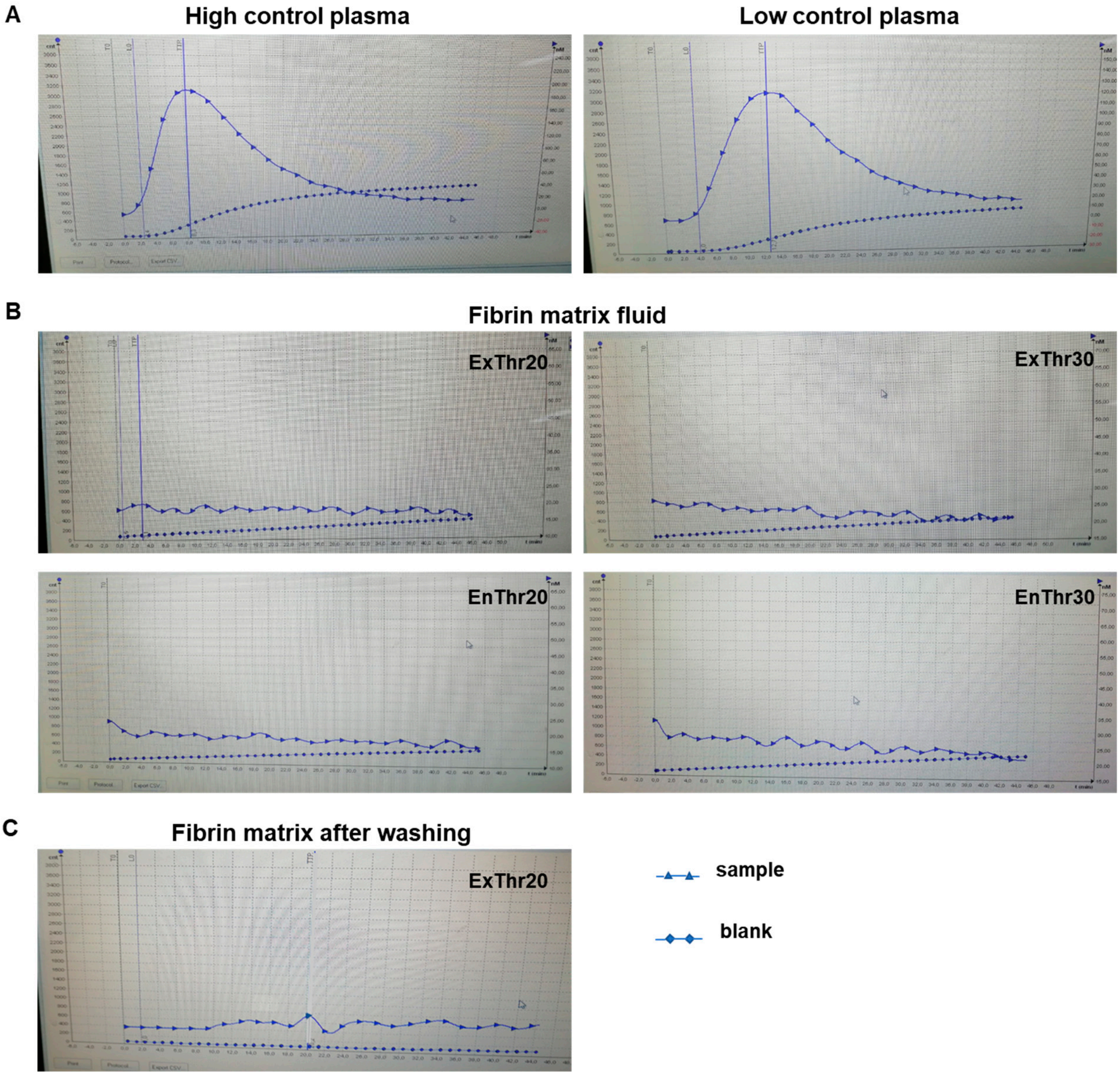

Supplementary Figure S3. Thrombin generation assay (TGA) profile of the (A) high control plasma and low control plasma (controls), (B) liquid that remained after the retraction of fibrin matrices prepared at fibrinogen concentrations of 20 and 30 mg/mL using endogenous (EnThr) and exogenous (ExThr) thrombin and (C) 0.9% sodium chloride solution in which ExThr 20 fibrin was incubated during 8 h after washing.
